# What length of after-school Learning time best promotes the development of non-cognitive ability in adolescents? Evidence from China

**DOI:** 10.3389/fpsyg.2025.1539137

**Published:** 2025-03-04

**Authors:** Huimin Sun, Haiping Xue

**Affiliations:** ^1^College of Educational Science, Xinjiang Normal University, Urumqi, Xinjiang, China; ^2^College of Education, Capital Normal University (CNU), Beijing, China

**Keywords:** after-school Learning time, school homework duration, extracurricular subject tutoring duration, non-cognitive ability, adolescents

## Abstract

We utilized a nationally representative dataset from the China Education Panel Survey (CEPS) and applied hierarchical linear models and quadratic models to analyze the impact of after-school Learning time on adolescents’ non-cognitive ability development, aiming to identify the optimal duration. Our results show that, first, the relationship between school homework duration and the development of openness, conscientiousness, extraversion, and agreeableness follows an inverted “U” curve, while its impact on neuroticism follows a “U” curve. Considering the five dimensions of non-cognitive ability development, the optimal daily school homework duration is recommended to be no more than 39.33 min. Second, the duration of extracurricular academic tutoring exhibits an inverted “U” relationship with openness, conscientiousness, extraversion, and agreeableness, with no significant effect on neuroticism. The optimal daily duration for extracurricular academic tutoring, based on four non-cognitive ability development dimensions, is recommended to be no more than 79.83 min. Third, the effect of after-school Learning time on the development of non-cognitive abilities varies based on the socioeconomic status (SES) of the family. Adolescents from low SES families tend to benefit from longer optimal durations of school homework and extracurricular academic tutoring compared to their peers from medium and high SES families.

## 1 Introduction

In the traditional framework of human capital theory, educational investment plays a central role in human capital development (Schultz, 1961; Becker, 1964; Hanushek and Woessmann, 2008). Within this framework, academic achievement and cognitive abilities are considered primary indicators of an individual’s capabilities, as they are seen to reflect the economic returns from education. However, human capital theory typically limits its focus to cognitive development, leading to a narrow conceptualization of “student ability” that is primarily based on exam scores and cognitive abilities, often neglecting non-cognitive abilities (Carneiro and Heckman, 2003). Existing research suggests that individual achievement is largely shaped by the personal abilities developed during adolescence. Both cognitive and non-cognitive abilities formed during this critical period have been shown to effectively predict a range of important adult outcomes, including educational attainment, job performance, income, health, and criminal behavior (Duckworth and Seligman, 2005; Currie, 2009; Lindqvist and Vestman, 2011). The new human capital theory emphasizes the importance of non-cognitive abilities, asserting that these abilities are crucial for an individual’s academic performance, employment, income, health, and social life (Zhou, 2015). Non-cognitive abilities, as essential abilities for adolescents to adapt to 21st-century society, not only influence academic performance and educational choices during adolescence but also impact labor market outcomes and quality of life in adulthood (Duckworth and Seligman, 2005; Heckman et al., 2006; Cunha et al., 2010; Glewwe et al., 2017). Thus, it is evident that both non-cognitive and cognitive abilities are equally important for adolescents’ future development (Cunha and Heckman, 2007; Kautz et al., 2014; OECD, 2018).

Several factors contribute to the development of non-cognitive abilities in adolescents. Among these, study duration is widely considered a crucial factor (Fredricks et al., 2004; Lavy, 2015). Carroll’s research proposed a model linking study time to learning outcomes, highlighting that study time is closely related to the development of individual abilities. According to his model, longer study time can directly improve cognitive outcomes, such as knowledge acquisition and comprehension abilities. This framework laid the foundation for subsequent studies examining the relationship between study time and student capabilities (Carroll, 1963). Additionally, research suggests that study duration not only influences the quantity of knowledge gained but also the quality of non-cognitive abilities, such as perseverance, self-discipline, and emotional regulation (Heckman et al., 2006; Lavy, 2015). Furthermore, time spent on after-school assignments contributes to the development of non-cognitive abilities, including responsibility and self-discipline (Cooper et al., 2006; Trautwein, 2007).

Existing literature on the impact of study time on non-cognitive outcomes provides a strong theoretical and empirical foundation for our study. However, does extended study time necessarily lead to the comprehensive development of students’ cognitive and non-cognitive abilities? This question warrants further investigation. In traditional Chinese culture, well-known proverbs such as “The sea of knowledge is boundless; hard work is the vessel” and “Diligence can make up for a lack of talent” reflect the belief that more study time will result in better academic outcomes for adolescents. Consequently, in practical education, the most common approach to fostering adolescents’ ability development has been to increase homework duration or enroll children in extracurricular tutoring classes. This has led much of the existing research to focus on the relationship between school homework duration, extracurricular academic tutoring, and adolescents’ cognitive abilities (Bray, 2013; Wang et al., 2014; Xu, 2020). However, in developing countries, particularly China, research on the causal relationship between study time and non-cognitive abilities remains relatively scarce. The available literature on this subject primarily stems from developed countries, with findings suggesting that an appropriate study duration can enhance non-cognitive abilities such as responsibility and self-discipline (Fredricks et al., 2004; Duckworth and Seligman, 2005). However, studies also caution that excessive study time may have negative consequences, such as increased psychological stress and feelings of frustration, which can impair emotional regulation and the overall learning experience (Heckman et al., 2006; Trautwein, 2007; Kalenkoski and Pabilonia, 2012). These results prompt an important reflection: while an appropriate study duration can positively support the development of non-cognitive abilities, excessive study time may have the opposite effect, leading to negative emotions and stress. Therefore, finding a balance between study time and the development of students’ non-cognitive abilities has become a critical issue that must be addressed in both educational research and practice.

At the theoretical level, German psychologist Ebbinghaus introduced the “overlearning effect” in his 1885 book *On Memory*. He proposed that while a moderate amount of learning is essential for ability development, excessive learning beyond a reasonable threshold may have negative consequences. Overlearning not only consumes more time and energy but may also lead to issues such as distracted attention, increased fatigue, and physical and mental exhaustion, ultimately resulting in a decline in abilities (Ebbinghaus, 1913). This theory provides a crucial framework for understanding the relationship between study time and ability development. Building on this foundation, this paper examines the current patterns of after-school Learning time investment among Chinese middle school students, aiming to address the following questions: Does after-school Learning time enhance adolescents’ non-cognitive abilities, or does overlearning hinder their development? What is the ideal duration of school homework and extracurricular academic tutoring for middle school students? Is there an optimal range for study time? To explore these questions, this paper will utilize data from the China Education Panel Survey (CEPS) and incorporate the Big Five personality theory to assess adolescents’ non-cognitive abilities, providing a comprehensive analysis of the relationship between after-school Learning time and the development of non-cognitive abilities. Additionally, building on the overlearning effect, this paper will calculate the optimal duration of school homework and extracurricular academic tutoring for middle school students. The study’s results aim to offer a foundation for improving the ability development of Chinese adolescents and inform the formulation of scientifically grounded educational policies.

## 2 Literature review and hypotheses

### 2.1 School homework duration and students’ non-cognitive abilities

After-school homework is a vital component of school education, often considered an effective tool for consolidating classroom knowledge and improving academic performance. In recent years, however, educational research has shifted its focus toward examining the impact of after-school homework on students’ non-cognitive abilities, such as self-efficacy, perseverance, time management, and emotional regulation. Non-cognitive abilities play a crucial role in students’ long-term development, contributing not only to academic success but also to career achievements and social adaptation. A well-balanced approach to after-school homework duration can offer students additional learning opportunities while simultaneously fostering the development of these essential skills (Cooper et al., 2006). Studies have shown that after-school homework requires students to complete tasks within a set timeframe, which helps cultivate time management skills. Zimmerman emphasized that students need to plan their time, set priorities, and monitor progress while completing homework, behaviors that enhance self-regulation abilities. These skills are valuable not only in academics but also in future career development. Additionally, by completing homework, students consolidate classroom knowledge and experience a sense of accomplishment, thereby boosting their self-efficacy (Zimmerman, 2002). Bandura highlighted that self-efficacy is a key factor influencing behavior choices, effort levels, and persistence. A moderate amount of homework offers students opportunities for practice and feedback, helping to strengthen their confidence in their abilities (Bandura, 1997). Moreover, challenging homework tasks foster grit, as perseverance is a critical predictor of long-term success. By overcoming obstacles and remaining focused on complex assignments, students develop persistence (Duckworth and Seligman, 2005). While moderate homework has a positive effect on the development of non-cognitive abilities, excessive homework can negatively affect students’ emotional wellbeing and motivation. Cooper found that excessive homework can lead to increased stress, sleep deprivation, and decreased interest in learning, which in turn undermines the development of non-cognitive abilities. Furthermore, while moderate homework helps develop time management skills and responsibility, excessive homework can result in anxiety and lower self-esteem (Cooper et al., 2006). Pope also noted that high-intensity homework may reduce adolescents’ social and rest time, hindering their social–emotional development (Pope, 2001).

Based on these observations, this study proposes the first research hypothesis.

*Hypothesis 1*: There is an inverted “U”-shaped relationship between homework time and non-cognitive abilities.

### 2.2 Extracurricular subject tutoring duration and students’ non-cognitive abilities

In recent years, extracurricular academic training (such as tutoring and subject-specific coaching) has experienced rapid growth, particularly in East Asia and some Western countries. While this type of training is primarily designed to improve academic performance, its impact on students’ non-cognitive abilities (such as self-efficacy, stress resilience, and social skills) has increasingly become a focus of academic research. Some studies suggest that moderate levels of extracurricular training can help students develop more efficient learning strategies, thereby enhancing self-efficacy (Bandura, 1997). For example, in math tutoring, setting incremental goals and providing feedback on problem-solving can help students gradually build confidence in their ability to tackle challenging problems. Additionally, personalized guidance from instructors may reinforce goal-oriented behaviors (Zimmerman, 2002). Some extracurricular programs, particularly those that involve repeated practice and mock exams, may help students adapt to high-pressure environments and develop greater stress resilience. However, “over-training” may lead to emotional exhaustion, goal-oriented training may undermine students’ intrinsic motivation for learning, leading to a decline in interest (Deci and Ryan, 2000; Suter, 2016). Larson emphasized that adolescents require unstructured activities (such as free play or club participation) to develop social skills. However, excessive extracurricular training can reduce the time available for such activities, limiting opportunities for peer interaction (Larson, 2001).

Therefore, this study proposes the second research hypothesis.

*Hypothesis 2*: There may be an inverted “U”-shaped relationship between the duration of extracurricular academic tutoring and adolescents’ non-cognitive abilities.

### 2.3 The moderating effect of socioeconomic status (SES)

After-school assignments and off-campus academic tutoring are the two primary forms of extracurricular learning, both extensively studied for their impact on academic performance. However, the influence of these activities on non-cognitive abilities—such as self-efficacy, perseverance, and emotional regulation—is highly complex and significantly moderated by socio-economic status (SES). SES profoundly affects students’ participation in, and outcomes from, extracurricular learning through mechanisms such as resource distribution, cultural capital, and family support. Regarding resource support, empirical studies show that high-SES families can offer quality resources, such as quiet study spaces, smart learning devices, and personalized tutoring, which help students efficiently complete tasks and reduce learning stress (Zhao, 2024). In contrast, low-SES families, due to limited resources, may face environmental distractions and insufficient support, potentially leading to suboptimal learning outcomes during homework and tutoring (Sirin, 2005). In terms of parental involvement, high-SES parents tend to use a “Guided Participation” approach, helping students understand learning goals through questioning and discussion (Lareau, 2018). This method aids in developing students’ critical thinking and intrinsic motivation, indirectly strengthening their non-cognitive abilities (Zhao, 2024). By comparison, low-SES parents, constrained by time or knowledge, are more likely to engage in supervisory involvement, which may unintentionally trigger parent–child conflicts when they cannot provide effective assistance, undermining the positive impact of extracurricular learning (Hoover-Dempsey et al., 2001). Additionally, high-SES families often incorporate extracurricular learning into educational planning through “cultural matching,” viewing tutoring as part of the “elite development path” and thereby reducing students’ anxiety regarding their goals (Bourdieu, 1986).

Based on this analysis, this study proposes the third research hypothesis.

*Hypothesis 3*: The impact of extracurricular learning time on non-cognitive abilities is moderated by SES.

## 3 Data and variables

### 3.1 Data

In this study, we used the 2014–2015 academic year database from the China Education Panel Survey (CEPS), a large-scale, longitudinal survey project that provides a representative analysis of the influence of family, school, community, and macro-social structures on individual educational outcomes. The survey also explores how educational outcomes shape an individual’s life trajectory. This project was designed and conducted by the China National Survey Research Center (NSRC) at Renmin University of China.

The China Education Panel Survey (CEPS), launched with the 2013–2014 academic year as its baseline, begins with two cohorts: the first-grade students in junior high school (Seventh Grade) and the third-grade students in junior high school (Ninth Grade). Stratified by average education levels and the proportion of mobile populations, the survey randomly selected 28 county-level units (including counties, districts, and cities) across China as survey sites. The survey was school-based, and within these selected units, 112 schools and 438 classes were randomly chosen for participation. All students in the selected classes were included in the baseline survey, totaling approximately 20,000 students. This project uses questionnaires as its main data collection tool. Surveys are administered to all participating students, their parents or guardians, class and subject teachers, and school principals. The questionnaire covers a broad range of topics, including students’ basic information, life experiences, physical and mental health, parent–child interactions, in-school learning, extracurricular activities, relationships with teachers and classmates, social development, and family member details. It also gathers parents’ basic information, lifestyles, family educational environment, parental investment in education, community environment, views on school education, interactions with teachers, and expectations for their children’s education. In addition, comprehensive cognitive ability tests and basic personality assessments are conducted for students, and key examination results (such as mid-term exams, high school entrance exams, and college entrance exams) are collected. Plans are also in place to conduct health and physical examinations, collect biomedical indicators, and employ various methods and technologies to comprehensively gather high-quality data.

Official reports for the CEPS, such as results, assessment frameworks, user guides, background survey questionnaires, databases, and relevant information, are available on the official website: http://www.cnsda.org/index.php?r=projects/view&id=61662993.

### 3.2 Variables

#### 3.2.1 Outcome variables

The outcome variable in this study is students’ non-cognitive abilities. Although there is no universally accepted method for measuring non-cognitive abilities in the academic field, the Big Five Inventory (BFI) is widely recognized (Goldberg, 1992; Costa and McCrae, 1999). Developed by John and Srivastava, the BFI assesses five personality dimensions: extraversion, agreeableness, conscientiousness, neuroticism, and openness (John and Srivastava, 1999). The inventory consists of 46 items scored on a 5-point Likert scale (1 = strongly disagree, 5 = strongly agree). Scores for each dimension are calculated as the average of all relevant items, with higher scores indicating stronger personality traits. As a result, the Big Five Personality structural model, rooted in personality psychology, has been widely adopted in both domestic and international research (York et al., 2008; Guha, 2015). The data for this study are derived from the China Education Panel Survey (CEPS) database, which was designed, implemented, and released by the China Survey and Data Center (NSRC) at Renmin University of China. Before the re-release of the data, the reliability and validity of the questionnaire were assessed, and it passed both tests. Additionally, the Big Five personality model scale, widely used in the China Education Panel Survey, has demonstrated strong reliability and validity, supported by a substantial body of literature (Fang and Cao, 2021; Zhou and Liu, 2022; Sun, 2024). The measurement tools employed in this study are based on these established studies, which have consistently reported excellent reliability and validity in measuring the five dimensions of non-cognitive abilities.

The empirical analysis in this study will be conducted using the Big Five Personality structural model. The measurement items for each personality dimension are standardized and aggregated to compute scores. Higher scores reflect stronger non-cognitive abilities in adolescents. A brief description of the questionnaire items corresponding to each dimension is outlined below. (1) Openness represents an individual’s traits, including imagination, curiosity, receptiveness to new experiences, and mental agility (John and Srivastava, 1999; Guha, 2015). The CEPS asks students whether they agree with the following statements: “I can easily engage in conversation with others,” “If my approach to handling a situation is incorrect, I will try to think of alternative methods to solve it,” “Even under tough and difficult situations, I can maintain composure,” and “I generally have confidence in tasks that I will complete.” Response options included completely disagree = 1; somewhat disagree = 2; somewhat agree = 3; completely agree = 4. (2) Conscientiousness refers to a dimension of an individual’s traits, including diligence, reliability, and punctuality (John and Srivastava, 1999; Guha, 2015). CEPS asks students whether they agree with the following self-descriptions: “Even if I feel a little unwell or have other reasons to stay home, I will still try my best to go to school,” “Even if it’s a subject I don’t like, I will do my best,” “Even if it takes a long time to complete the homework, I will still keep trying,” and “I can persist in my interests and hobbies.” The response options included completely disagree = 1; somewhat disagree = 2; somewhat agree = 3; completely agree = 4. (3) Extroversion refers to the dimension of an individual’s traits such as being outgoing, helpful, and skilled in social interactions (John and Srivastava, 1999; Smillie, 2013). CEPS asks students about school life and whether they agree with the following statements: “I often participate in school or class activities,” “I find it easy to get along with others,” “Most of my classmates are friendly to me,” and “The class I am in has a good atmosphere.” The response options included completely disagree = 1; somewhat disagree = 2; somewhat agree = 3; completely agree = 4. (4) Agreeableness refers to the dimension of an individual’s traits such as sympathy, compliance, humility, and trust (John and Srivastava, 1999; Tackett et al., 2013). CEPS asks students if they can do the following in the past year: “Be keen to help the elders,” “Observe order and queue up voluntarily,” and “Being sincere and friendly to others.” The response options included never = 1; occasionally = 2; sometimes = 3; often = 4; always = 5. (5) Neuroticism is a dimension of an individual’s psychological characteristics, including anxiety, depression, and emotional fluctuations, typically with a negative impact (John and Srivastava, 1999; Shiner, 2019). CEPS asks students about their feelings in the past seven days: “In the past seven days, I felt depressed,” “In the past seven days, I felt of melancholy,” ‘In the past seven days, I felt unhappy,” “In the past seven days, I felt life had no meaning,” and “In the past seven days, I felt sad.” The options included never = 1; rarely = 2; sometimes = 3; often = 4; always = 5. We reverse-coded the options as follows: always = 1; often = 2; sometimes = 3; rarely = 4; never = 5. The scores for Openness, Conscientiousness, Extraversion, Agreeableness, and Neuroticism were standardized. Higher scores in Openness, Conscientiousness, Extraversion, and Agreeableness indicate greater competence, whereas higher scores in Neuroticism reflect lower emotional stability.

#### 3.2.2 Independent variables

The key independent variable in this study is the duration of after-school Learning, which comprises two components: school homework duration and extracurricular subject tutoring duration. These components are recalibrated and then combined, with a higher numerical value indicating a longer overall after-school Learning duration.

The questionnaire items related to school homework duration are described as follows: CEPS asked students two questions: (1) “On weekdays, how long do you usually spend on homework assigned by your schoolteachers each day?,” (2) “On weekends, how long do you usually spend on homework assigned by your schoolteachers each day?.” After recalibration, the options included: 0 h = 1; 1.5 h = 2; 2.5 h = 3; 3.5 h = 4; 4 h or more = 5.

The questionnaire items related to extracurricular subject tutoring duration are described as follows: CEPS also asked students two questions: (1) “On weekdays, how long do you usually spend on extracurricular tutoring classes (related to school subjects) each day?,” (2) “On weekends, how long do you usually spend on extracurricular tutoring classes (related to school subjects) each day?.” After recalibration, the options included: 0 h = 1; 1.5 h = 2; 2.5 h = 3; 3.5 h = 4; 4 h or more = 5.

#### 3.2.3 Control variables

We consider the following student, school and family covariate variables: gender (male = 0, female = 1); whether the student is an only child (yes = 0, no = 1); family socioeconomic status (SES), which is derived from a principal component factor analysis of three variables: family economic status, highest parental education level, and parental highest occupational prestige. The SES variable is standardized. When measuring parents’ years of schooling and occupation type, we use the information from either the father or the mother, whichever is higher; school location (rural areas = 1, semi-urban = 2, urban = 3); school quality (poor = 1, average = 2, good = 3). For detailed explanations of relevant variables, see Table 1.

**Table 1:** Description of variables.

Variable type	Variable name	Description of variables
Independent variables	School homework duration	Continuous variable
	Extracurricular subject tutoring duration	Continuous variable
Outcome variables	Openness	Continuous variable
	Conscientiousness	Continuous variable
	Extraversion	Continuous variable
	Agreeableness	Continuous variable
	Neuroticism	Continuous variable
Control variables	Gender	Male = 0, female = 1
	The only child	Yes = 0, no = 1
	Family socioeconomic status (SES)	Low = 1, medium = 2, high = 3
	School location	Rural areas = 1, semi-urban = 2, urban = 3
	School quality	Poor = 1, average = 2, good = 3

## 4 Methods

### 4.1 Multilevel linear models

Multilevel linear models are well-suited for hierarchical analyses, as they allow for the effective use of information at different levels. These models decompose the factors influencing students’ non-cognitive abilities across various relevant levels, resulting in more accurate estimates and more meaningful interpretations. Developed to overcome the limitations of traditional statistical techniques in handling multilevel structured data, multilevel linear models prevent potential misinterpretation of results. They are ideal for conducting thorough and appropriate analyses of commonly encountered nested data (Osborne and Neupert, 2013).

In analyzing the impact of post-homework time on students’ non-cognitive abilities, a multilevel linear model is employed. This approach is necessary because, in the CEPS, student samples are nested within different schools, and variations in teaching quality and student-teacher ratios exist across schools. To account for the nested structure of the data, a two-level linear model (HLM2) is used to examine the effects of both student-level and school-level variables on students’ non-cognitive abilities. The model construction begins with estimating the null model to determine the percentage of variance in the dependent variable explained by between-group differences and assess its significance. HLM2 is then constructed based on these initial findings.

#### 4.1.1 Null model

This model decomposes the total variance in non-cognitive ability levels of adolescents into two levels: individual students and between schools. It is primarily used to investigate whether there are significant differences in non-cognitive ability levels among schools. The model is as follows:

(1) $$\mathrm{Student}-\mathrm{level}:Y_{ij}=\beta_{0j}+r_{ij},r_{ij}\sim N\left(0,\delta^{2}\right)$$

(2) $$\mathrm{School}-\mathrm{level}:\beta_{0j}=\gamma_{00}+\mu_{0j},\mu_{oj}\sim N\left(0,\tau_{00}\right)$$

In this context, Y_ij_ represents the non-cognitive ability levels of the i-th student in the j-th school, encompassing dimensions such as extraversion, agreeableness, neuroticism, openness, and conscientiousness. β_0j_ represents the average non-cognitive ability levels of students in school j, γ_00_ represents the overall non-cognitive ability level across all students, μ_0j_ represents the random effects between schools, δ^2^represents the student-level differences in non-cognitive abilities, and τ_00_ represents the differences in non-cognitive abilities between schools.

#### 4.1.2 Full model

The full model is constructed by incorporating student-level and school-level variables into the null model, as follows:

(3) $$\begin{array}{l} \mathrm{Student}-\mathrm{level}:Y_{ij}=\beta_{0j}+\beta_{1j}{ST}_{i}+\beta_{2j}{ET}_{i}+\beta_{3j}X_{i}\\ \;+\beta_{4j}F_{i}+r_{ij},r_{ij}\sim N\left(0,\delta^{2}\right) \end{array}$$

(4) $$\mathrm{School}-\mathrm{level}:\beta_{0j}=\gamma_{00}+\gamma_{01}S_{i}+\mu_{0j},\mu_{0j}\sim N\left(0,\tau 00\right)$$

From the student-level perspective, Y_ij_ represents the non-cognitive ability outcomes of the i-th student in the j-th school. β_0j_ represents the non-cognitive ability outcomes of school j. ST_i_ represents the school homework duration variable, ET_i_ represents the extracurricular tutoring time variable, F_i_ represents the family socioeconomic status variable, and X_i_ represents individual student variables. r_ij_ represents the random effects among students. From the school-level perspective, S_i_ represents school-level variables, and μ_0j_ represents the random effects among schools.

### 4.2 Quadratic model

Based on the theories of overlearning effect and cognitive load theory, and to verify whether the impact of after-school Learning time on non-cognitive abilities in adolescents follows an inverted “U”-shaped curve, the model includes squared terms for homework time and extracurricular tutoring time. Here, ST^2^_i_ represents the school homework duration variable, ET^2^_i_ represents the extracurricular tutoring time variable. The quadratic model is constructed as follows:

(5) $$\begin{array}{l} \mathrm{Student}-\mathrm{level}:Y_{i}=\beta_{0j}+\beta_{1}T_{i}+\beta_{2}{{ST}^{2}}_{i}+\beta_{3}{{ET}^{2}}_{i}\\ \;+\beta_{4}X_{i}+\beta_{5}F_{i}+\varepsilon_{i},\varepsilon_{i}\sim N\left(0,\delta^{2}\right) \end{array}$$

(6) $$\mathrm{School}-\mathrm{level}:\beta_{0i}=\gamma_{00}+\gamma_{01}S_{i}+\mu_{0j},\mu_{0j}\sim N\left(0,\tau_{00}\right)$$

## 5 Main results

### 5.1 The null model

In this study, we used IBM SPSS Statistics 26 for data processing, statistical analysis, and model estimation. All regression analyses, including grouped regressions and robustness checks, were conducted in SPSS 26, ensuring consistency and reproducibility in data processing. To examine the factors influencing students’ non-cognitive ability development concerning after-school Learning time, we employed a multilevel linear model. Based on the null models (Equation 1) and (Equation 2), we first constructed an null model without explanatory variables to analyze the sources of variance in non-cognitive ability development. As shown in Table 2, the intraclass correlation coefficients (ICCs) for Openness, Conscientiousness, Extraversion, Agreeableness, and Neuroticism are 0.103, 0.134, 0.297, 0.167, and 0.113, respectively. The ICCs exceed the threshold of 0.059, supporting the application of a multilevel linear model for analysis.

**Table 2:** Null model—decomposition of sources of student non-cognitive skill differences.

RE		VC	ICC	*p*
Openness	Intercept	0.103	0.103	0.000
	Level 1	0.951		
Conscientiousness	Intercept	0.134	0.134	0.000
	Level 1	0.939		
Extraversion	Intercept	0.297	0.297	0.000
	Level 1	0.864		
Agreeableness	Intercept	0.167	0.167	0.000
	Level 1	0.992		
Neuroticism	Intercept	0.113	0.113	0.000
	Level 1	0.969		

Notes: ICC less than 0.059 indicates low intragroup correlation, 0.059–0.138 bits moderate intragroup correlation, and greater than 0.138 is high intragroup correlation.

### 5.2 Multilevel analysis

Based on the full models (Equation 3) and (Equation 4) as well as the quadratic models (Equation 5) and (Equation 6), we constructed hierarchical linear models to analyze the impact of after-school learning time on adolescents’ non-cognitive ability development. The specific results are shown in Table 3. First, the duration of school homework has a significant negative impact on Neuroticism, while significantly promoting the development of other non-cognitive abilities. All results are significant at the 0.01 and 0.001 levels, indicating that increasing school homework duration suppresses Neuroticism and fosters the positive development of non-cognitive traits such as Openness, Conscientiousness, Extraversion, and Agreeableness. Second, the duration of extracurricular academic tutoring positively affects Openness, Conscientiousness, Extraversion, and Agreeableness, all at the 0.001 level, with no effect on Neuroticism. These findings suggest that increasing the duration of extracurricular tutoring enhances the positive development of adolescents’ non-cognitive abilities in these areas.

**Table 3:** The impact of after-school learning time on adolescent non-cognitive ability.

Variable name	Non-cognitive abilities				
	Openness	Conscientiousness	Extraversion	Agreeableness	Neuroticism
Student level					
SHD	0.043** (0.012)	0.143*** (0.012)	0.06*** (0.011)	0.110*** (0.011)	−0.068*** (0.012)
ESTD	0.176*** (0.024)	0.092*** (0.024)	0.142*** (0.023)	0.071** (0.023)	0.035 (0.024)
SHD^2^	−0.067*** (0.008)	−0.099*** (0.008)	−0.069*** (0.008)	−0.048*** (0.008)	0.052*** (0.008)
ESTD^2^	−0.049** (0.006)	−0.042* (0.006)	−0.048*** (0.006)	−0.04* (0.006)	0.001 (0.007)
Male = 0 (Yes)	0.029 (0.021)	−0.095 *** (0.021)	−0.080*** (0.020)	−0.165*** (0.020)	−0.095*** (0.021)
Only children = 0 (Yes)	0.037 (0.024)	0.019 (0.024)	−0.013 (0.024)	0.014 (0.024)	−0.073** (0.025)
SES = 1 (Low)	−0.080* (0.038)	−0.138*** (0.038)	−0.158*** (0.037)	−0.125*** (0.038)	0.162*** (0.039)
SES = 2 (Medium)	−0.089** (0.032)	−0.144*** (0.032)	−0.101*** (0.031)	−0.120*** (0.031)	0.092** (0.032)
School level					
School quality = 1 (Poor)	−0.109 (0.071)	−0.167** (0.072)	−0.032 (0.115)	−0.075 (0.085)	0.014 (0.067)
School quality = 2 (Average)	−0.114* (0.057)	0.021 (0.058)	−0.032 (0.094)	−0.043 (0.070)	−0.090* (0.054)
School location = 1 (Rural areas)	−0.036 (0.045)	−0.060 (0.046)	−0.014 (0.074)	−0.125** (0.055)	−0.008* (0.043)
School location = 2 (Semi-urban)	0.023 (0.062)	0.022 (0.063)	0.030 (0.103)	0.025 (0.076)	0.008 (0.058)
Intercept	0.013** (0.052)	0.052*** (0.052)	0.052*** (0.064)	0.008*** (0.055)	0.235***(0.051)
Intergroup variance	0.029	0.031	0.105	0.051	0.024
Intragroup variance	0.915	0.897	0.837	0.864	0.942
*p*-value	0.000	0.000	0.000	0.000	0.000
*N*	8,681	8,645	8,681	8,741	8,654

Notes: (i) SHD represents school homework duration, and SHD^2^ represents the squared term of school homework duration. (ii) ESTD represents extracurricular subject tutoring duration, and ESTD^2^ represents the squared term of extracurricular subject tutoring duration. (iii) Robust standard errors in parentheses. (iv) ***, **, and * indicate statistical significance from zero at the 1, 5, and 10 percent levels.

Table 3 also show that the quadratic term of school homework duration significantly negatively affects adolescents’ Openness, Conscientiousness, Extraversion, and Agreeableness in both cognitive and non-cognitive abilities, while having a significant positive effect on Neuroticism. These findings suggest that the relationship between school homework duration and the development of adolescents’ cognitive and non-cognitive abilities follows a non-linear pattern. Specifically, the effect of school homework duration on Neuroticism is U-shaped, meaning that as the amount of homework increases, the negative impact on Neuroticism reaches a minimum point, after which the effect weakens. Additionally, it can be inferred that homework duration follows an inverted U-shaped effect on the other four non-cognitive dimensions, suggesting that a moderate amount of school homework promotes the development of Openness, Conscientiousness, Extraversion, and Agreeableness. However, beyond a certain threshold, excessive homework duration has a negative impact. Furthermore, the results show that the quadratic term of extracurricular subject tutoring duration significantly negatively affects adolescents’ Openness, Conscientiousness, Extraversion, and Agreeableness, with no effect on Neuroticism. It is speculated that extracurricular tutoring time has an inverted U-shaped effect on adolescents’ non-cognitive abilities: a certain duration of tutoring promotes the development of Openness, Conscientiousness, Extraversion, and Agreeableness, but exceeding a threshold result in a negative impact. Hence, H1 and H2 is verified.

To further validate the inverted U-shaped relationship, a heterogeneous analysis was conducted by dividing the sample into three groups based on students’ family socioeconomic status (SES): low SES, middle SES, and high SES. The estimation results in Table 4 show that the effect of after-school Learning time on students’ non-cognitive abilities across different socioeconomic backgrounds is consistent with the overall findings presented in Table 3. Specifically, for all SES groups, the duration of school homework exhibits a U-shaped effect on adolescents’ Neuroticism and an inverted U-shaped effect on Openness, Conscientiousness, Extraversion, and Agreeableness. The duration of extracurricular subject tutoring has no significant effect on Neuroticism but demonstrates an inverted U-shaped effect on Openness, Conscientiousness, Extraversion, and Agreeableness. These results further confirm the existence of a critical point in the effect of after-school Learning time on adolescents’ non-cognitive abilities, which optimizes their non-cognitive performance. Hence, H3 is verified.

**Table 4:** The impact of after-school learning time on adolescent non-cognitive ability (different family socioeconomic status).

Variable name	Non-cognitive abilities				
	Openness	Conscientiousness	Extraversion	Agreeableness	Neuroticism
SES = 1 (Low)	(1)	(2)	(3)	(4)	(5)
SHD	0.096*** (0.019)	0.182*** (0.018)	0.105*** (0.019)	0.142*** (0.018)	−0.036** (0.018)
ESTD	0.057*** (0.059)	0.064* (0.049)	0.176*** (0.050)	0.082*** (0.048)	0.079 (0.048)
SHD^2^	−0.022*** (0.014)	−0.097*** (0.013)	−0.072*** (0.014)	−0.037** (0.013)	0.049*** (0.013)
ESTD^2^	−0.052*** (0.015)	−0.019* (0.015)	−0.024* (0.015)	−0.028* (0.014)	−0.009 (0.014)
Control variables	Yes	Yes	Yes	Yes	Yes
Intercept	0.235*** (0.072)	0.289*** (0.070)	0.331*** (0.087)	0.260*** (0.075)	0.001** (0.071)
Intergroup variance	0.030	0.029	0.102	0.047	0.030
Intragroup variance	0.915	0.859	0.902	0.851	0.849
*p*-value	0.000	0.000	0.000	0.000	0.000
*N*	2,778	2,763	2,785	2,799	2,766
SES = 2 (Medium)	(6)	(7)	(8)	(9)	(10)
SHD	0.029* (0.017)	0.125*** (0.017)	0.040** (0.016)	0.099*** (0.017)	−0.068*** (0.017)
ESTD	0.177*** (0.032)	0.141*** (0.032)	0.144*** (0.031)	0.096** (0.032)	0.022 (0.033)
SHD^2^	−0.059***(0.012)	−0.097*** (0.012)	−0.062*** (0.011)	−0.06*** (0.011)	0.053*** (0.012)
ESTD^2^	−0.056** (0.009)	−0.037* (0.009)	−0.034* (0.008)	−0.014* (0.009)	−0.001 (0.009)
Control variables	Yes	Yes	Yes	Yes	Yes
Intercept	0.058*** (0.055)	0.212*** (0.058)	0.030*** (0.067)	0.101*** (0.060)	0.126** (0.057)
Intergroup variance	0.021	0.033	0.103	0.053	0.022
Intragroup variance	0.921	0.921	0.834	0.886	0.972
*p*-value	0.000	0.000	0.000	0.000	0.000
*N*	4,453	4,438	4,446	4,484	4,448
SES = 3 (High)	(11)	(12)	(13)	(14)	(15)
SHD	0.047* (0.034)	0.109** (0.034)	0.018** (0.031)	0.046* (0.032)	−0.015* (0.037)
ESTD	0.144** (0.051)	0.044* (0.051)	0.117** (0.046)	0.116** (0.048)	0.030 (0.054)
SHD^2^	−0.077*** (0.022)	−0.093*** (0.022)	−0.061* (0.020)	0.017* (0.021)	0.034* (0.024)
ESTD^2^	−0.08* (0.012)	−0.012* (0.012)	−0.034** (0.011)	−0.048* (0.012)	0.005 (0.013)
Control variables	Yes	Yes	Yes	Yes	Yes
Intercept	0.218*** (0.108)	0.030*** (0.106)	0.138*** (0.103)	0.056** (0.102)	0.449 (0.114)
Intergroup variance	0.035	0.018	0.067	0.026	0.020
Intragroup variance	0.886	0.893	0.721	0.816	1.023
*p*-value	0.000	0.000	0.000	0.000	0.000
*N*	1,450	1,444	1,450	1,458	1,440

Notes: (i) SHD represents school homework duration, and SHD^2^ represents the squared term of school homework duration. (ii) ESTD represents extracurricular subject tutoring duration, and ESTD^2^ represents the squared term of extracurricular subject tutoring duration. (iii) Robust standard errors in parentheses. (iv) ***, **, and * indicate statistical significance from zero at the 1, 5, and 10 percent levels.

### 5.3 Instrumental variable approach

This study employs the instrumental variable approach to address endogeneity concerns, including reverse causality, in the empirical regression analyzing how socioeconomic status (SES) moderates the effect of after-school learning duration on adolescents’ non-cognitive development.

The instrumental variable for socioeconomic status (SES) is derived from the CEPS parent questionnaire, specifically the item “whether the family receives a subsistence allowance.” Family eligibility for a subsistence allowance is correlated with SES, while having minimal direct influence on adolescents’ non-cognitive abilities, supporting its validity as an instrumental variable. Table 5 presents the first-stage regression results of the instrumental variable “whether the family receives a subsistence allowance” (subsistence allowance) on openness, conscientiousness, extraversion, agreeableness, and neuroticism. The estimated coefficient of the instrumental variable is positive and statistically significant. The Kleibergen-Paap rk LM and Kleibergen-Paap rk Wald F statistics confirm that the instrumental variable (subsistence allowance) successfully passes both the under-identification and weak instrument tests, verifying its effectiveness. The second-stage regression incorporating the instrumental variable demonstrates that SES significantly influences multiple dimensions of adolescents’ non-cognitive abilities. These results indicate that the empirical findings remain robust and credible even after addressing endogeneity concerns.

**Table 5:** Addressing endogeneity in the moderating role of socioeconomic status (SES) on the relationship between after-school learning time and adolescents’ non-cognitive abilities (an instrumental variable approach).

Variable name	First-stage	Second-stage	First-stage	Second-stage	First-stage	Second-stage	First-stage	Second-stage	First-stage	Second-stage
	SES	Openness	SES	Conscientiousness	SES	Extraversion	SES	Agreeableness	SES	Neuroticism
Subsistence allowance	0.436***(0.019)		0.437***(0.019)		0.434*** (0.019)		0.434*** (0.019)		0.437*** (0.019)	
SES		0.665** (0.201)		1.121*** (0.203)		1.197*** (0.221)		0.929*** (0.178)		−2.774*** (0.461)
Kleibergen-Paap rk LM		386.361 [0.000]		384.767 [0.000]		382.768 [0.000]		386.215 [0.000]		385.933 [0.000]
Kleibergen-Paap rk Wald F		517.62 {16.38}		517.31 {16.38}		510.467 {16.38}		515.103 {16.38}		518.62 {16.38}
*N*	9,055	9,055	9,016	9,016	9,051	9,051	9,118	9,118	9,024	9,024

(i) ***, **, and * indicate statistical significance from zero at the 1, 5, and 10 percent levels. (ii) () values represent robust standard errors, [] values represent *p*-values, and {} values represent the critical values at the 10% level for the Stock-Yogo weak identification test.

### 5.4 Measurement of the optimal duration of after-school learning

The estimation results in Tables 3, 4 indicate that the relationship between after-school Learning time and adolescents’ non-cognitive abilities—openness, conscientiousness, extraversion, agreeableness—follows an inverted U-shaped curve, except for neuroticism, which exhibits a U-shaped curve. These findings suggest that longer after-school Learning time does not necessarily result in better development of non-cognitive abilities. Instead, there is a turning point at which the positive effects of after-school Learning time on non-cognitive development begin to decline. The optimal duration of after-school Learning time for adolescents can be determined by calculating the inflection point of the quadratic function in each model.

The results in Table 6 show that, for the full sample of students, a moderate duration of after-school homework promotes the development of non-cognitive abilities such as openness, conscientiousness, extraversion, and agreeableness while reducing neuroticism. However, exceeding a critical threshold reverses these benefits, negatively affecting adolescents’ non-cognitive abilities. For school homework, the optimal durations for promoting adolescents’ non-cognitive abilities are 19.25 min for openness, 43.33 min for conscientiousness, 26.09 min for extraversion, 68.75 min for agreeableness, and 39.23 min for neuroticism. Homework within these time thresholds positively contributes to the development of adolescents’ non-cognitive abilities. For extracurricular subject tutoring, the optimal durations are 107.75 min for openness, 65.71 min for conscientiousness, 92.61 min for extraversion, and 53.25 min for agreeableness. Tutoring within these time frames is more beneficial for fostering the development of these dimensions of adolescents’ non-cognitive abilities. Moreover, considering openness, conscientiousness, extraversion, agreeableness, and neuroticism as five interconnected dimensions of non-cognitive abilities, the average values of these indicators can be used to estimate the optimal durations for after-school learning. The ideal time for school homework is 39.33 min, while the optimal duration for tutoring is 79.83 min.

**Table 6:** Optimal study duration (in minutes).

		Openness	Conscientiousness	Extraversion	Agreeableness	Neuroticism	Average
Full sample	SHD	19.25	43.33	26.09	68.75	39.23	39.33
	ESTD	107.75	65.71	92.61	53.25	·	79.83
Low SES	SHD	11.9	56.28	43.75	115.14	22.04	49.82
	ESTD	130.91	101.05	220	87.86	·	134.96
Medium SES	SHD	14.75	38.65	19.38	49.5	38.49	32.16
	ESTD	94.83	114.32	127.09	70.24	·	101.61
High SES	SHD	18.31	35.16	8.85	·	13.23	15.11
	ESTD	54	110	81.18	72.5	·	79.42

(i) SHD represents school homework duration, and SHD^2^ represents the squared term of school homework duration. (ii) ESTD represents extracurricular subject tutoring duration, and ESTD^2^ represents the squared term of extracurricular subject tutoring duration. (iii) · indicates statistical insignificance.

Furthermore, the critical points for optimal after-school Learning duration vary across different student groups. Teenagers from low SES families have longer optimal after-school learning durations, both for school homework and extracurricular tutoring, compared to those from middle and high SES families. Specifically, for teenagers from low SES families, the greatest developmental effect on non-cognitive abilities occurs when the school homework duration is less than 49.82 min, while extracurricular academic tutoring has the greatest impact when limited to 134.96 min. For teenagers from middle SES families, a school homework duration of 32.16 min is most beneficial for the development of non-cognitive abilities, while extracurricular academic tutoring lasting up to 101.61 min is most effective. To optimize the effect of after-school learning on the development of non-cognitive abilities, the ideal school homework duration for teenagers from high SES families is 15.11 min, with an optimal duration for extracurricular tutoring of 79.42 min.

## 6 Mechanism conclusion

Against the backdrop of the China “double reduction” policy, investigating the optimal duration of after-school learning to better promote students’ non-cognitive ability development holds great significance, and thus the following findings are revealed:

Firstly, the relationship between school homework duration and the development of adolescents’ non-cognitive abilities exhibits a nonlinear pattern. Specifically, school homework duration has a “U-shaped” effect on the development of adolescents’ neuroticism and an “inverted U-shaped” relationship with the other four dimensions of non-cognitive abilities. Integrating the inflection points of the five non-cognitive ability developments, the optimal average daily school after-school homework duration should not exceed 39.33 min. According to Skinner’s reinforcement theory, school homework extends classroom teaching effectively, making the teaching and learning process more complete. Students can absorb and understand classroom knowledge by completing school homework. Additionally, completing school homework helps cultivate good study habits and self-discipline, which is conducive to fostering students’ independent learning and innovative abilities, thereby promoting the development of adolescents’ non-cognitive abilities to some extent (Goldstein, 1960; Bas et al., 2017). Furthermore, this conclusion also confirms the hypothesis of overlearning effect, namely, when the homework time exceeds the total cognitive resources of students, cognitive overload may occur, resulting in an uneconomical state and diminishing marginal returns, which gradually decreases learning effectiveness and is detrimental to the improvement of students’ non-cognitive abilities. Therefore, longer school homework duration is not necessarily better for adolescents, and attention should be paid to the marginal effect of homework. The research results of this study also indicate that the China “double reduction” policy stipulating that the homework time for junior high school students should not exceed 1.5 h is scientifically reasonable, as it not only does not reduce students’ non-cognitive abilities, but also has enhancing effects.

Secondly, the duration of extracurricular subject tutoring exhibits an inverted U-shaped relationship with the four dimensions, and it has no significant effect on adolescents’ neuroticism. Considering the inflection points of the four dimensions of non-cognitive ability development, the optimal daily average extracurricular tutoring time should not exceed 79.83 min. Most parents choose subject-based extracurricular classes primarily to improve their children’s academic performance, while some parents lack the ability to guide their children’s homework, thus having to opt for extracurricular tutoring classes (Guo et al., 2020; Zheng et al., 2020). Currently, considering that some parents and students still have demands, the current “double reduction” policy has not abolished extracurricular subject training but is regulating the industry to ensure its healthy development. Adolescents participating in extracurricular tutoring not only enhance their sense of learning efficiency during the process of improving their grades but also enhance their ability to adapt to society, such as self-expression, cooperation, interpersonal communication, and communication abilities (Deci and Ryan, 2000; Zins and Elias, 2007; Johnson and Johnson, 2009), thereby contributing to the improvement of non-cognitive abilities to some extent (Duckworth and Seligman, 2005). Additionally, the strict time regulations on subject-based extracurricular tutoring under the “double reduction” policy are partly due to the consideration of the duration and intensity of extracurricular tutoring, which may affect children’s physical and mental health. If the time is too long or the burden is too heavy, the rate of myopia among children will be higher, making them more prone to illness, and leading to more psychological health problems such as depression and anxiety (Buckley and Lee, 2021). Furthermore, extracurricular tutoring has a serious “crowding out” effect on students’ physical exercise and labor education time, and prolonged extracurricular tutoring will harm the development of other dimensions of non-cognitive abilities (Bray, 2013; Zheng et al., 2020; Zhang and Gao, 2023).

Thirdly, the impact of post-school learning time on the development of non-cognitive abilities varies among different socioeconomic backgrounds. The inflection points of school homework duration and extracurricular subject tutoring duration for adolescents from low SES families are 49.82 min and 134.96 min, respectively, both higher than those of middle SES and high SES families. As the “Coleman Report” pointed out, families, especially parents, are the root cause of educational inequality, and schools maintain and strengthen the original differences brought about by family backgrounds (Coleman, 1968). Parents from low socioeconomic backgrounds generally lack cultural and economic capital. First, in terms of cultural capital, they lack sufficient time to accompany and care for their children, and their lower educational and cultural levels result in insufficient ability to guide their children’s academic work. Therefore, their children almost entirely rely on teachers for learning (Sullivan, 2001; Lareau, 2018). Compared to adolescents from families with higher socioeconomic status, they need more time to reinforce and understand classroom knowledge. The limited time for classroom teaching determines that the role and function of school homework are more obvious for adolescents from low socioeconomic backgrounds, which also verifies that within a certain range, school homework duration can weaken the unequal educational outcomes caused by differences in family socioeconomic status (Parcel and Dufur, 2001; Shi and Xue, 2022). Secondly, in terms of economic capital, after the marketization of education, the choice of educational products for children is largely constrained by the economic capital possessed by parents’ social class (Jin and Yang, 2015). Parents from lower socioeconomic backgrounds hope more to break through the shackles of social class and want their children to attend good universities, find good jobs, and lead a good life (Xiong, 2017). Under the dual mechanism of high educational expectations and limited economic capital, parents from lower socioeconomic backgrounds pay more attention to cost-effectiveness when choosing extracurricular tutoring for their children and are more inclined to choose large class tutoring classes. Therefore, adolescents need more tutoring time to fully leverage its effects.

## 7 Discussion

Numerous studies have highlighted the relationship between study duration, academic outcomes, and cognitive abilities, particularly in the context of developing countries. However, research examining the causal link between study time and non-cognitive abilities remains relatively scarce, often constrained by data limitations or methodological challenges, and lacks robust empirical evidence. The primary objective of this study is to evaluate the impact of after-school Learning time on adolescents’ non-cognitive abilities and explore the mechanisms underlying this relationship. By addressing these gaps, this study aims to provide a clearer understanding of the causal connection between study duration and non-cognitive abilities and identify potential pathways, thereby contributing valuable theoretical and empirical insights to inform the development of evidence-based educational policies in China.

The findings of this study support the scientific validity and rationale behind China’s “Double Reduction” policy, particularly its limitations on adolescents’ homework time. This policy emphasizes strict control over homework volume and incorporates tiered strategies to address the diverse learning needs of students at different academic levels. Teaching practices should adopt a stratified approach, tailored to the specific needs of students, and allocate homework time judiciously to identify optimal durations for different student profiles, thereby promoting more effective development of non-cognitive abilities. Furthermore, China’s burden-reduction policies should encourage educational institutions to shift toward diversified and holistic educational approaches. Training organizations should focus on designing programs that stimulate creativity, foster critical thinking, and enhance physical fitness, all of which contribute to the advancement of innovative educational models. Lastly, the research underscores the importance of family education as a key component of a child’s educational ecosystem, with parental involvement being vital for students’ development. Thoughtful and scientifically informed parental engagement can significantly improve students’ academic performance while also supporting their social and non-cognitive abilities development in a healthy, balanced manner.

This study makes the following key contributions to the literature: First, it provides novel causal inferences regarding the impact of study time on adolescents’ non-cognitive abilities, based on empirical analysis. Furthermore, it expands the research scope beyond traditional cognitive outcomes to include non-cognitive ones, offering new theoretical and empirical insights into the holistic development of Chinese students. Second, the study uses nationally representative data and applies hierarchical linear modeling (HLM) for analysis. This approach controls for the effects of both student-level and school-level variables, while also enabling a more comprehensive examination of the interactions between these factors and their influence on students’ non-cognitive abilities. This design ensures the precision of the results and enhances the depth of interpretation. Third, the study explores various potential mechanisms underlying the causal relationship between after-school Learning time and adolescents’ non-cognitive abilities. This exploration helps deepen the understanding of how study duration influences the development of non-cognitive abilities, laying the groundwork for future research in this area.

While this study makes significant theoretical and practical contributions, we must also recognize its limitations, mainly stemming from the constraints of the sample database. First, this research focuses on Chinese students, and due to differences in culture, education systems, and policy environments, the findings may not be easily generalized to other countries or populations. Future research should investigate the relationship between study duration and non-cognitive abilities in diverse cultural contexts to identify patterns with broader applicability. Second, due to limitations in data resources, we were unable to fully explore potential mediating mechanisms and confounding factors. Future studies may consider incorporating more diverse and extensive datasets or using advanced analytical techniques to further examine the causal relationship between after-school Learning duration and non-cognitive abilities. In subsequent research, we aim to address these limitations by obtaining more suitable data and employing advanced methodologies.

## Data Availability

The datasets presented in this study can be found in online repositories. The names of the repository/repositories and accession number(s) can be found below: http://www.cnsda.org/index.php?r=projects/view&id=61662993.
